# Lifetime prevalence of intimate partner violence against women in an urban Brazilian city: A cross-sectional survey

**DOI:** 10.1371/journal.pone.0224204

**Published:** 2019-11-14

**Authors:** Tendai Kwaramba, Jinny J. Ye, Cyrus Elahi, Joseph Lunyera, Aline Chotte Oliveira, Paulo Rafael Sanches Calvo, Luciano de Andrade, Joao Ricardo Nickenig Vissoci, Catherine A. Staton

**Affiliations:** 1 Duke Global Health Institute, Duke University, Durham, North Carolina, United States of America; 2 Division of Emergency Medicine, Department of Surgery, Duke Medical Center, Durham, North Carolina, United States of America; 3 Centro Universitário Ingá, Maringá, PR, Brazil; 4 Universidade Estadual de Maringá, Maringá, PR, Brazil; 5 Division of Global Neurosurgery and Neuroscience, Department of Neurosurgery, Duke Medical Center, Durham, North Carolina, United States of America; Christiana Care/University of Delaware, UNITED STATES

## Abstract

**Background:**

Intimate partner violence is a global health burden that disproportionately affects women and their health outcomes. Women in Brazil are also affected by interpersonal violence. We aimed to estimate the lifetime prevalence of three forms of interpersonal violence against women (IPVAW) and to identify sociodemographic factors associated with IPVAW in one urban Brazilian city.

**Methods:**

Using a cross-sectional design, we interviewed women aged ≥18 years in the urban Brazilian city, Maringá, who currently have or have had an intimate partner. The 13-item WHO Violence Against Women instrument was used to ask participants about their experiences with intimate partner violence, categorized into psychological, physical and sexual violence. We estimated associations between IPVAW and sociodemographic characteristics using generalized linear models.

**Results and conclusions:**

Of the 419 women who were enrolled and met inclusion criteria, lifetime prevalence of IPVAW was 56%. Psychological violence was more prevalent (52%) than physical (21%) or sexual violence (13%). Twenty-eight women (6.4%) experienced all three forms of IPVAW. Women were more likely to experience violence if they were employed, did not live with their partner or had 4 or more children. Educational level, household income, age and race were not significantly associated factors. Our findings highlight a high prevalence of IPVAW in a community in southern Brazil.

## Introduction

At some point in their lifetime, 1 in 3 women worldwide will have experienced intimate partner violence.[1] Intimate partner violence against women (IPVAW) is defined by the World Health Organization (WHO) as women’s self-reported experience of physical, sexual or psychological harm or threats of such harm at the hands of their intimate partners or ex-partners. The growing recognition of IPVAW as a prevalent global issue was informed by the WHO’s Multi-Country Study on Women’s Health and Domestic Violence Against Women. The WHO Multi-Country Study found a lifetime physical violence ranging from 13% to 61%, sexual violence from 6% to 59%, and psychological violence from 21% to 90%.[1]

IPVAW has serious and negative social, medical and economic consequences for individuals and families.[2] IPVAW is associated with food insecurity,[3] lower birthweight of newborns,[4] delay in initiation of childcare,[5] and child maltreatment.[6] Previous work found that IPVAW is associated with sociodemographic characteristics, such as young age, lower education, and other health behaviors, including alcohol use.[7] Because of the correlation between sociodemographic factors and individual health, IPVAW is recognized as a human rights violation and an important public health issue.[8]

Brazil criminalized violence against women in 2006.[9] The 2006 legislation, commonly referred to as the Maria da Penha Law,[9] also expanded a network of services (i.e. police, justice system) and promoted research studies, program implementation, and educational campaigns.[10]Furthermore, Brazil codified the mandatory reporting of IPVAW by healthcare providers in 2003[11] and created a standardized notification form in Brazil’s national health database, *Sistema de Informação de Agravos de Notificação* (SINAN), [12] in 2009.[13]

In Brazil, the estimated lifetime prevalence of physical violence throughout the country is as high as 16.7% and 2.4% for sexual violence.[14] This 2017 estimate was higher than previous nationwide estimates from the Brazilian National Alcohol and Drugs Survey in 2012 estimating physical violence at 6.3%.[15] Prevalence estimates varies not only over time but also by region and by type of violence. In the southern state of Paraná, our region of interest, IPVAW clustered mostly around the southern part of the state with one cluster in the northern mesoregion. [16] In the southeastern city of São Paulo, lifetime prevalence of physical violence was 27.2% compared to the rural northeastern province of Zona da Mata de Pernambuco (33.8%). In both these areas, physical violence was more prevalent than sexual violence (10.1% in São Paulo and 14.3% in Zona da Mata de Pernambuco). [17] Physical violence was found to be particularly high (30%) in the southeast city of Rio de Janeiro in women with children. [18]

While previous studies have estimated prevalence of IPVAW and associated sociodemographic characteristics in Brazil, these studies have focused on either physical violence [19] or sexual violence[16]. Only one study in Brazil has examined psychological aspects of violence in addition to physical and sexual violence. [20] This study in the state of São Paulo found the lifetime prevalence of IPVAW in any form was 55.7% with 53.8% psychological, 32.2% physical, and 12.4% sexual. To have a more encompassing scope of IPVAW in Brazil, the current study estimates the prevalence of three forms of IPVAW using a cross-sectional design in the urban city of Maringá. Our second aim was to identify victim sociodemographics associated with IPVAW.

## Materials and methods

### Brazilian health system

Brazil’s national health system, *Sistema Único de Saúde* (SUS), is a complex network of distinct but interconnected public and private services. Primary care services use a community-based and public health approach, primarily through the structure of family health teams and the primary care clinic, referred to as the Basic Health Unit. Health teams include a physician, a nurse, a nurse assistant, and community health agents who serve a geographical catchment of 600 to 1,000 households with no gaps or overlaps. Community health agents visit households periodically regardless of need or demand to collect health screening data, reach individuals lost to follow-up, and act as a bridge between primary care and public health.[21]

### Study setting

**Fig 1:**
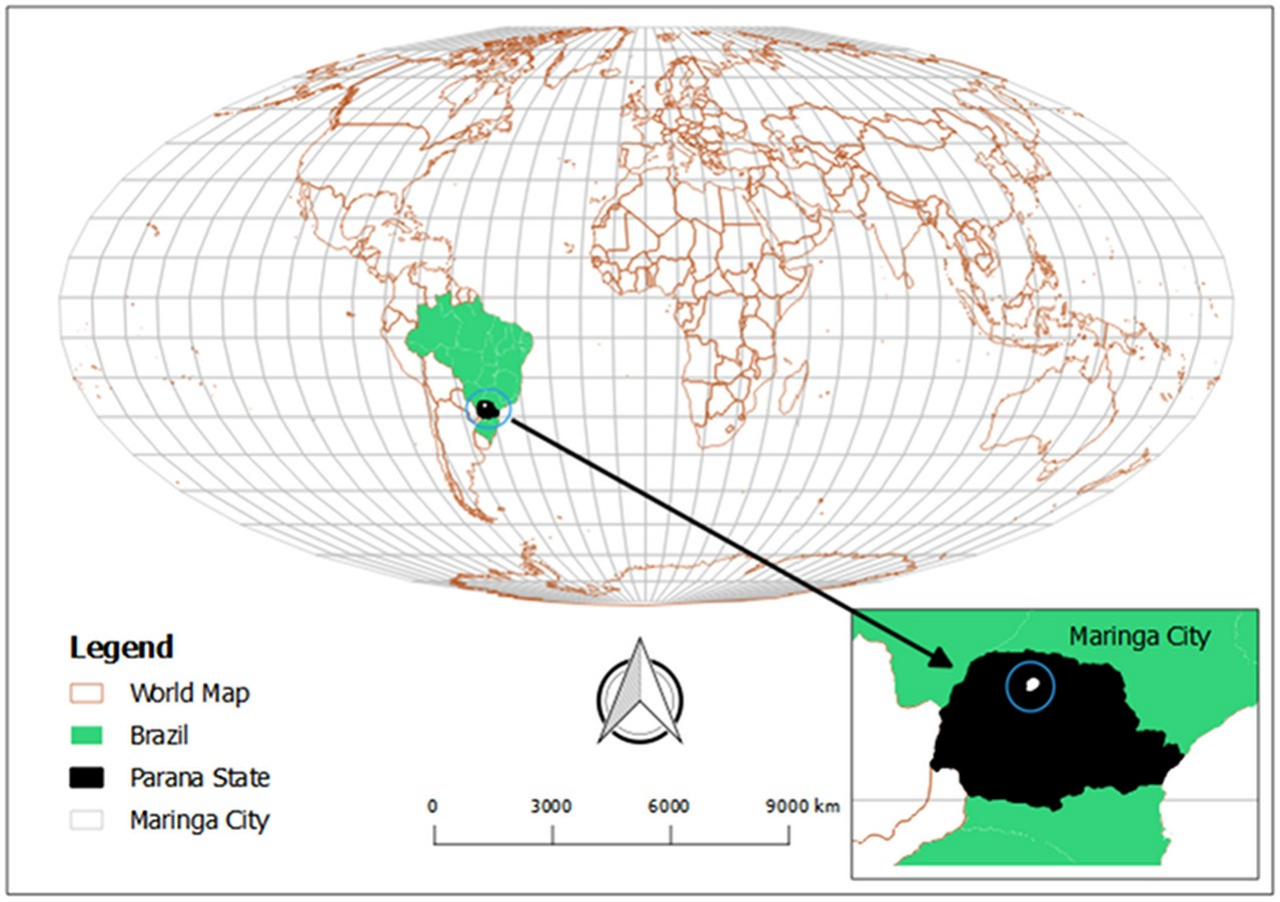
Location of study setting created from OpenStreetMap.org.

The target population lived in a municipality with a corresponding single health team within Maringá, an urban city in southern Brazil (Fig 1). Maringá is the third most populated city in the state of Paraná with 357,077 inhabitants in 2010 [22]. Forty-four percent of the inhabitants are females aged 18 years or older. The population is predominantly white (71% of the population), followed by 22% mixed race, 4% Asian, 3% black, and <1% indigenous. In regards to education, 3.3% of the population 18 years and older never went to school. [23]

### Study participants

Using a cross-sectional design, we used a convenience sample by visiting all households in a geographical catchment formed by one Basic Health Unit in October 2014 in Maringá, Brazil. The households were approached by the study team two times in the day (once in the morning and once in the afternoon). Participants were only interviewed once. However, a second visit was conducted if the interview was not possible at the first time. Households were not included if there were no women in the house to participate during any of both data collection visits, if the participant did not feel safe or secure to respond, or did not consent to participate in the study. The study team included the community health agent assigned as part of the existing health system prior to this study. Households ranged from a single women, couples and multiple generations. In each household, women at least 18 years of age, who currently or previously had an intimate partner, were invited to participate in the study. Our sample was composed of all the houses (N = 1,517) within the geographical catchment area with a target population of 1,748 women. A total of 435 women were enrolled in the study, but 16 had to be excluded due to incomplete responses to the survey. Our final enrollment sample size of 419 women was more than enough to be representative of the population of that community with 5% significance, an error margin of 0.01, and an estimated prevalence of IPVAW of 25% [21,22].

### Assessment instruments

Demographic data were collected using Brazil’s Ministry of Health standardized questionnaire on general women’s health. We collected data on participants’ age, race, education, family income, occupation, cohabitation with partner, and number of children. We used the WHO’s definition of IPVAW. [1] Our definition does not include abuse of female children, genital mutilation, violence perpetrated or condoned by the State, or violence occurring within the general community.

**Fig 2:**
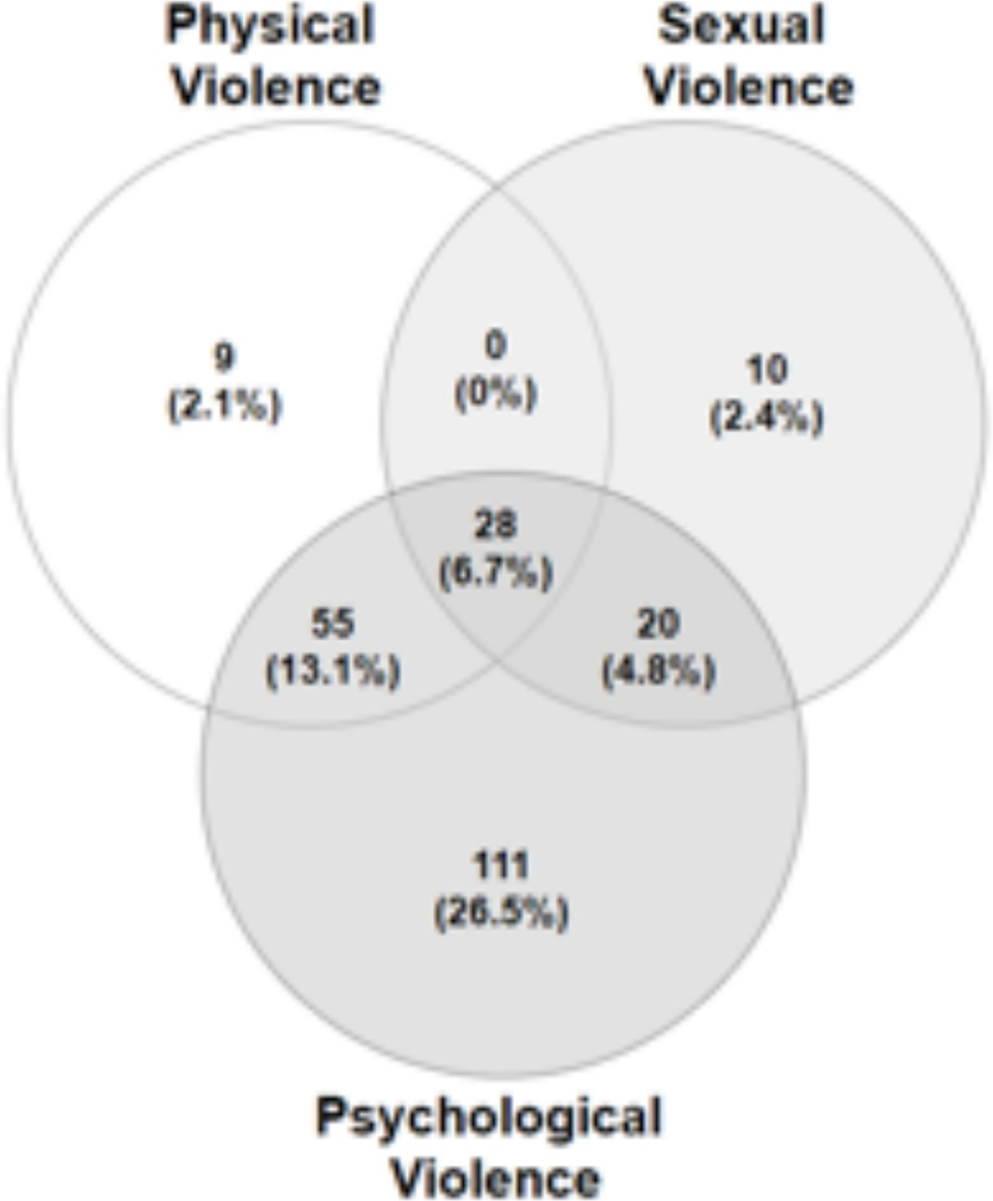
Prevalence of combinations of cases of lifetime psychological, physical and sexual violence.

The WHO Violence Against Women (VAW) instrument was administered to all participants face-to-face through a community health agent. The 13 items in the VAW instrument asked questions on psychological, physical and sexual violence to capture participants’ experiences with intimate partner violence (Fig 2).[17] This instrument was previously translated into Brazilian Portuguese and validated in Brazil.[24] Additionally, the instrument was pretested for flow, comprehensibility, and administrative ease among 20 women in Mandacaru, a neighborhood within the target community. No changes were made to the questionnaire after pretesting.

### Data collection

A team of researchers, including a community health agent, one psychology student, and one medical student, knocked door-to-door of sampled households during weekdays between 8:00 am and 5:00 pm to minimize contact with spouses. To address social desirability bias and ensure safety and confidentiality of respondents during the interview sessions, respondents used a code phrase at any time during the interview if they felt unsafe because of the presence or arrival of a third party (spouse, family members, or neighbors). When this happened, the interviewer used the Ministry of Health questionnaire on general women’s health to collect general demographic data. This general women’s health survey was previously standardized by the Brazilian Ministry of Health and is done periodically by the health team for health surveillance. If a male answered the door (father, partner, or son), he was asked to speak to women in the household for a study on women’s health. If no one answered the door at any of both visits, households were not revisited. Study personnel obtained verbal and written consent from participants prior to study enrollment. Our research team only performed the interviews with the women when they were alone. If a spouse, family member or neighbor would not leave the participants side, we completed the health survey and returned to the house another time. For women who experienced IPVAW, women were offered social work and psychology services as well as given standardized pamphlets from the women’s police station.

### Statistical analysis

Study personnel entered questionnaire data in a secure location shared only with the primary investigators. The data were checked for accuracy by two independent study personnel and then exported to STATA v.13 software (StataCorp., College Station, TX) for analyses. We excluded 16 observations due to missing data but the missing data were deemed to be at random. Lifetime prevalence estimates for IPVAW and selected sociodemographic characteristics of participants were calculated with 95% confidence intervals (CI). Prevalence estimates were not weighted because our sampling design allowed for a similar chance to capture most of the targeted community. We used generalized linear models to estimate the associations between IPVAW and the sociodemographic characteristics. Univariable and multivariable logistic regression models for lifetime psychological violence, physical violence, sexual violence and overall IPVAW were developed with the following predictors: age as a continuous variable, educational level and 3 socioeconomic status markers (income, occupation and number of children). Odds ratios (OR) for the exposure-outcome associations were estimated using a logit link, and a p-value of <0.05 was considered statistically significant. For continuous variables, median and interquartile ranges (IQR) were reported, and difference between groups were estimated using a Chi-squared test, Fisher’s Exact test, or the Wilcoxon-Mann-Whitney rank sum test.

We focused on the association between IPVAW and socioeconomic status (SES) of the women as indicators of social disadvantage and contextual stressors influencing individual behavior. The indicators of SES assessed were income, education, occupation, and number of children. Income levels were categorized based on the poverty line set by the Brazilian Institute of Geography and Statistics. Income levels are reported in this study as less than 1, 1 to 3, or greater than 3 times the poverty line. [25] Results were reported in accordance to the Strengthening the Reporting of Observational Studies in Epidemiology (STROBE) statement. [26] All data files are available from the Figshare database (https://doi.org/10.6084/m9.figshare.6193070.v1).

### Ethical statement

This project was approved by the Faculdade Ingá Institutional Review Board in Maringá, Paraná, Brazil (617.636) and the Duke University Institutional Review Board in Durham, North Carolina, USA (C0256 and C0257).

## Results

Of the 1,517 households with a target population of 1,748 eligible women, a total of 435 women were eligible and enrolled but 16 women were excluded due to missing data. Of the remaining 419 participants,218 (52%) were white, 129 (31%) mixed race, and 39 (9%) black. The median age of participants was 50.9 years (IQR: 40–63). Only 128 (31.4%) participants completed high school or higher levels of education. The majority (225; 54%) did not have paid work outside the home and 263 (64.6%) reported a monthly family income 1 to 3 times the poverty line.[25] The majority (311; 74.2%) were living with an intimate partner, and 323 (79.8%) had at least two children (Table 1).

**Table 1:** Sociodemographics of study participants.

	Overall, n (%)	Lifetime IPVAW, n (%)	No IPVAW, n (%)	p-value
**Age (n = 419)**				0.838
** 18–29 years old**	54 (12.89)	29 (12.45)	25 (13.44)	
** 30–39 years old**	49 (11.69)	26 (11.16)	23 (12.37)	
** 40–49 years old**	101 (24.11)	60 (25.75)	41 (22.04)	
** ≥50 years old**	215 (51.31)	118 (50.64)	97 (52.15)	
**Race (n = 419)**				0.1669
** White**	218 (52.03)	128 (54.94)	90 (48.39)	
** Mixed race**	129 (30.79)	73 (31.33)	56 (30.11)	
** Black**	39 (9.31)	18 (7.73)	21 (11.29)	
** Asian descent**	11 (2.63)	5 (2.15)	6 (3.23)	
** Indigenous**^**a**^	2 (0.48)	2 (0.86)	0	
** Unknown**	20 (4.77)	7 (3.00)	13 (6.99)	
**Education (n = 408)**				<0.001
** Pre-high school**	280 (68.63)	156 (68.12)	124 (69.27)	
** High school**	104 (25.49)	63 (27.51)	41 (22.90)	
** Post-high school**	24 (5.88)	10 (4.37)	14 (7.82)	
** Not reported**				
**Monthly household income (n = 407)**				0.735
** <1 times poverty line**	72 (17.69)	41 (18.14)	31 (17.13)	
** 1 to 3 times poverty line**	263 (64.62)	148 (65.49)	115 (63.54)	
** >3 times poverty line**	72 (17.69)	37 (16.37)	35 (19.34)	
**Occupation (n = 419)**				0.004
** Paid work**^**b**^	142 (33.89)	94 (40.34)	48 (25.80)	
** Unemployed**	225 (53.70)	109 (46.78)	116 (62.37)	
** Other**^**c**^	52 (12.41)	30 (13.45)	22 (11.83)	
**Living with partner (n = 419)**	311 (74.22)	159 (68.24)	152 (81.72)	0.003
**Number of children (n = 405)**				0.466
** 0–1**	82 (20.25)	43 (19.03)	39 (21.79)	
** 2–3**	228(56.30)	125 (55.31)	103 (57.54)	
** ≥4**	95 (23.46)	58 (25.66)	37(78.21)	

^a^Indigenous people of Brazil are of Amerindian descent

^b^Paid work includes work without a formal contract and self-employment

^c^Other may include but is not limited to unpaid internships or volunteering

Out of 419 participants, 233 women (56%) experienced at least one form of IPVAW in their lifetime. Of the three forms of IPVAW, psychological violence was the most prevalent (51.1%: 47.5–56.9%) followed by physical violence (21.9%: 17.6–25.3%) and sexual violence (13.9%: 10.4–16.9%). Many women reported a lifetime history of experiencing more than one form of IPVAW. Twenty-eight women (6.7%: 4.5–9.2%) experienced all three forms of IPVAW; 55 (13.1%) experienced both psychological and physical violence; 20 (4.8%) experienced psychological and sexual violence. No participant experienced physical and sexual violence without psychological violence (Fig 2).

**Fig 3:**
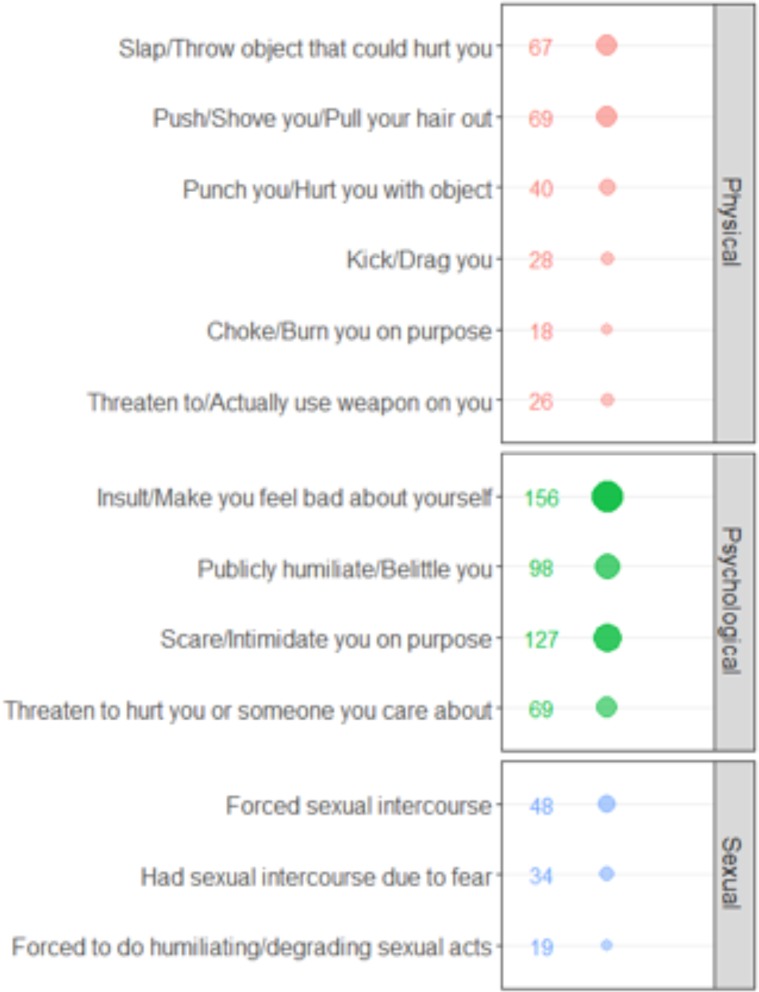
Lifetime prevalence of psychological, physical, and sexual violence.

Among women who experienced psychological violence from their intimate partners, many reported being insulted or made to feel bad about oneself (156; 37.2%), 127 women reported feeling scared or intimidated (30.3%), and 98 women reported being publicly humiliated (23.4%). Sixty-nine (16.5%) women reported that their partners threatened them or someone they care about (Fig 3). The most prevalent forms of physical violence reported in this study were pushing, shoving and/or pulling hair out (69; 16.5%), slapping or throwing an object at subject (67; 16.0%), and punching or hurting subject with an object (40; 9.5%). The most prevalent form of sexual violence was forced sexual intercourse (48; 11.5%).

### Correlates of psychological, physical, & sexual forms of IPVAW

In unadjusted models, women who had no paid work outside the home were significantly less likely to experience either psychological, physical or sexual violence compared to women with paid work. This association remained consistent in an adjusted model. This model showed women without paid work were about two times less likely to experience psychological, physical, or sexual forms of IPVAW than women who had paid work (Table 2). Women without paid work were also about two times less likely to experience all forms of IPVAW compared to women with paid work. Women who had four or more children were about three times more likely to experience physical forms of IPVAW, but not psychological, sexual, or all forms of IPVAW than women who had fewer than two children. Older women were more likely to experience sexual forms of IPVAW. In unadjusted models, the majority of women who encountered IPVAW did not complete high school, had an income 1–3 times the poverty line, and had at least 2 children. However, these associations were not significant in fully adjusted models (Table 2). Age and race were not significantly associated with lifetime experience in IPVAW in the multivariable model.

**Table 2:** Lifetime prevalence of intimate partner violence against women and sociodemographic associations in an adjusted multivariable model.

	Overall (n = 419)	Psychological violence	Physical violence	Sexual violence
	OR (95% CI)	OR (95% CI)	OR (95% CI)	OR (95% CI)
**Age**				
** 18–29 years old**	--	--	--	--
** 30–39 years old**	0.96 (0.22–1.39)	0.83 (0.36–1.87)	0.89 (0.30–2.62)	1.27 (0.19–10.40)
** 40–49 years old**	1.37 (0.64–2.96)	1.12 (0.53–2.40)	1.52 (0.60–4.11)	3.90 (0.93–27.04)
** ≥50 years old**	1.03 (0.48–2.21)	0.95 (0.44–2.03)	0.71 (0.26–2.00)	3.36 (0.77–23.84)
**Educational level**				
** Pre-high school**	--	--	--	--
** High school**	1.39 (0.81–2.40)	1.35 (0.79–2.31)	1.14 (0.59–2.17)	1.80 (0.79–4.06)
** Post-high school**	0.59 (0.22–1.50)	0.59 (0.22–1.50)	0.49 (0.10–1.66)	0.46 (0.02–2.68)
**Income**				
** <1 times poverty line**	--	--	--	--
** 1 to 3 times poverty line**	1.07 (0.60–1.90)	1.13 (0.64–2.00)	0.90 (0.47–1.77)	0.80 (0.38–1.74)
** >3 times poverty line**	0.89 (0.43–1.87)	0.88 (0.42–1.82)	1.07 (0.44–2.56)	0.58 (0.18–1.76)
**Occupation**				
** Paid work**^**a**^	--	--	--	--
** Unemployed**	0.40 (0.25–0.64)	0.45 (0.28–0.71)	0.48 (0.27–0.84)	0.52 (0.26–1.06)
** Other**^**b**^	0.61 (0.30–1.26)	0.62 (0.30–1.28)	0.64 (0.28–1.50)	0.77 (0.29–2.04)
**Number of children**				
** 0–1**	--	--	--	--
** 2–3**	1.1 (0.63–1.93)	1.13 (0.65–1.96)	1.34 (0.67–2.81)	1.54 (0.62–4.4)
** 4+**	1.42 (0.67–3.03)	1.36 (0.65–2.86)	2.58 (1.06–6.53)	1.91 (0.64–6.31)
**Cohabitation**				
** Living with partner**	--	--	--	--
** Not living with partner**	2.28 (1.33–3.96)	2.21 (1.31–3.76)	3.18 (1.76–5.78)	2.52 (1.28–4.96)

^a^Paid work includes work without a formal contract and self-employment

^b^Other may include but is not limited to unpaid internships or volunteering

## Discussion

In this cross-sectional study in the southern Brazilian city of Maringá, we found that lifetime prevalence of at least one form of IPVAW was 56%. Compared to the cross-sectional WHO Multi-Country Study on Women’s Health and Domestic Violence, this prevalence is higher than the lifetime prevalence of at least one form of IPVAW in the southeast city of São Paulo (46.4%) and the rural northeast region of Zona da Mata de Pernambuco (54.2%).[27] Despite being an urban area, Maringá interestingly has a higher lifetime prevalence similar to a rural region. Our finding suggests that this community is particularly vulnerable to IPVAW.

This study also supports prior work on the distribution between three types of violence that women experience: lifetime psychological violence as the most common, physical and lastly, sexual.[28] Our study is further consistent with prior work in other countries in that few of our participants experienced physical and sexual violence without also experiencing psychological violence. Thus, future interventions may benefit from addressing psychological violence in addition to other forms of IPVAW.

Women in this community who experienced IPVAW have sociodemographic characteristics similar to and different from other women in the world who experience IPVAW. Women in this community who experience IPVAW are more likely to be employed, have more children, and not cohabiting with their partner. Employment status has had mixed associations in studies throughout the world [29–32] and not associated with IPVAW in northeastern Brazil. [33] The unclear association of women’s employment status may be due to the complex interplay of socioeconomics and gender norms. Women who are employed may violate normative gender roles, leading the relationship to have more psychological stress. This strain, in turn, may lead to more violence in order to exert control over the relationship. However, independent income for women may also provide resources to prevent and end violent relationships. The theoretical complexity of women’s employment status and its implication on relationship dynamics mirrors the inconsistent associations found in previous studies.[32,34]

In this study, having 4 or more children was found to be associated with lifetime experience of physical violence. While studies have not established why having more children is associated with more violence between parents, it can be surmised that parenting could create economic insecurities as well as feelings of stress or jealousy within the violent partner. [6, 35] Additionally, previous studies have established the prevalence of co-occurring child maltreatment and IPVAW in the U.S, [6, 36] suggesting violence is not isolated to partners but may be the result of underlying harmful family dynamics. Because of this relationship, pediatricians and other health care providers have a unique position to screen for IPVAW. [37]

Interestingly, cohabitation with one’s partner was found to be protective factor of IPVAW. This finding is in contrast to a previous studies throughout the world, including in Brazil. [38–42] It would be of interest to explore the role of marital status and cohabitation in IPVAW in this community.

Surprisingly, these IPVAW prevalence findings were not associated with educational level and income. These findings contrast previous studies that have shown lower levels of education and per capita income as risk factors for IPVAW [19,20,38,43,44]. Our study suggests that while economic disadvantages may create stressors and vulnerabilities that contribute to the experience of IPVAW [28, 45], focusing primarily on poverty reduction strategies may not address other underlying causes of IPVAW in this setting. These underlying causes may include patriarchal constructs, perceived threats to dominance, permeation of normative power dynamics within intimate relationships, childhood exposure to IPVAW or other precipitating factors, such as substance or alcohol use, stress or feelings of jealousy [19, 43, 46].

The strengths of this study were having community health agents accompany our researchers and communicate with participants in the national language of Portuguese. This already established connection provided participants with a sense of familiarity and safety about personal information. The involvement of community health agents further developed a sense of rapport and grew existing health networks within the community. Participants’ openness during the study is suggested by a low rate of missing survey data.

The findings presented in this study should be interpreted in the context of some limitations. Underreporting may still exist because of fear of retaliation, of being discovered, shame, or women who have died from intimate partner violence.[10, 47] Convenience sampling may have produced unmeasured bias and a sample not representative of the population, especially in a heterogeneous population. [48] Using multistage cluster sampling methods in future studies may result in a more representative sample to allow for generalizability of results. [19] The median age of our participant population was 51 years and were unemployed, which may reflect the time survey data were collected (between the 8am and 5pm on weekdays) to minimize contact with working spouses. However, this also meant that working women may have been inadvertently excluded due to the sampling method. This study is also limited by the use of cross-sectional data that highlight associations but not causations. Our findings were also not tracked in time to evaluate associations between IPVAW and changes related to participants’ age, relationships with their partners, and societal changes. Lastly, the questionnaire was aimed at women who survived violence. Self-reporting behavior influences the data collected and may still lead to underreporting.

The findings from this study demonstrates IPVAW is a problem affecting the majority of women in this community. The sociodemographic associations are not entirely consistent with other studies in Brazil and other LMIC. Larger studies are needed to understand why this community is particularly vulnerable to IPVAW.
